# Supplementary material for: Low recruitment drives the decline of red porgy (Pagrus pagrus) along the southeast USA Atlantic coast: Inferences from fishery-independent trap and video monitoring
Source: PLoS One. 2023 Jul 5;18(7):e0286078. doi: 10.1371/journal.pone.0286078 (PMC10321639; doi:10.1371/journal.pone.0286078)
Supplement: Attachment — Submitted filename: Responses to Reviewers.docx [file pone.0286078.s001.docx]

Additional Editor Comments:

The reviewer suggested that there is an error (on page 18, line 373) - the wrong figure is referred to - This does indeed appear to be an error which should be changed. The other comments from the reviewer should be viewed as suggestions that the authors are free to ignore as these are not material to whether the manuscript meets PLOS ONE's publication criteria.

**Thank you so much for catching this error – we have now changed the text to read “Fig. 2” on line 373.**

My evaluation of the manuscript:

The authors use a large long-term (30-year) data set to explore a number of questions regarding red porgy that are pertinent to the fishery and species in general. The methods they use are appropriate, their analyses are statistically rigorous, and their conclusions are supported by their results. The manuscript is well written and (once the error identified by the reviewer has been addressed) appears to meet all PLOS ONE criteria for publication.

**Thank you very much for your review and editorial work on our manuscript.**

Reviewer #1:

An interesting paper that investigated the ways in which red porgy relative abundance and mean size varied in the southeast United States Atlantic coast. Based on the increase in the average length of individuals and the decline of juveniles over the time series, the authors conclude that the red porgy is currently experiencing low recruitment, which would be one of the reasons for the decline in species abundance. The manuscript has a considerable amount of data (20,920 trap samples; 13,081 videos) over a 32-year time series. The data is well analyzed and interpreted and would make a solid contribution to fishing science, so I suggest that the manuscript be accepted for publication.

**We thank Reviewer #1 for their thoughtful review of our manuscript.**

Throughout the text I found only a small mistake, on page 18, line 373, it mentions figure 1, but I believe that the sentence is about figure 2.

**Thank you so much for catching this error – we have now changed the text to read “Fig. 2” on line 373.**

Below I suggest two small questions that are up to the authors to incorporate into the discussion or not, if they believe it is necessary. In my opinion, a biological issue that I think might be important in debating recruitment failure was not raised in the discussion. What are the possible effects in relation to depletion that could be intensified with protogynous hermaphroditism, in terms of sex ratio and change in average size. With larger individuals currently found, wouldn't a higher proportion of males be expected in this population? I believe that the authors should raise this information in the literature and elucidate these speculations in the discussion about recruitment.

**We had mentioned on lines 625-627 that recruitment overfishing could be occurring in red porgy due to sperm limitation from skewed sex ratios, but the reviewer rightly suggests that males could actually become more common than females as average sizes become larger. Thus, we now include “or egg” in the following sentence on line 627: “…due to Allee effects or egg or sperm limitation from skewed sex ratios [46,47].”**

Another issue that caught my attention was that observed catches were higher in the northern part of the study area and in deeper waters. Is there the possibility of a stock shift to colder and deeper regions in response to environmental changes? Is there information about the species above North Carolina that could support this discussion?

**In our manuscript, we quantified the current spatial distribution of red porgy in the region. However, it is beyond the scope of this manuscript to describe how red porgy might shift their spatial distribution in the face of future environmental changes in the region, which was not a part of our analyses. Based on analyses from the NEFSC trawl survey, which samples in the northeast USA down to central North Carolina, red porgy are only very rarely caught north of North Carolina.**
